# Unsupervised Exemplar-Domain Aware Image-to-Image Translation

**DOI:** 10.3390/e23050565

**Published:** 2021-05-02

**Authors:** Yuanbin Fu, Jiayi Ma, Xiaojie Guo

**Affiliations:** 1 College of Intelligence and Computing, Tianjin University, Tianjin 300350, China; yuanbinfu@tju.edu.cn; 2 Electronic Information School, Wuhan University, Wuhan 430072, China; jyma2010@gmail.com

**Keywords:** image-to-image translation, neural style transfer, unsupervised learning, generative adversarial network

## Abstract

Image-to-image translation is used to convert an image of a certain style to another of the target style with the original content preserved. A desired translator should be capable of generating diverse results in a controllable many-to-many fashion. To this end, we design a novel deep translator, namely exemplar-domain aware image-to-image translator (EDIT for short). From a logical perspective, the translator needs to perform two main functions, i.e., feature extraction and style transfer. With consideration of logical network partition, the generator of our EDIT comprises of a part of blocks configured by shared parameters, and the rest by varied parameters exported by an exemplar-domain aware parameter network, for explicitly imitating the functionalities of extraction and mapping. The principle behind this is that, for images from multiple domains, the content features can be obtained by an extractor, while (re-)stylization is achieved by mapping the extracted features specifically to different purposes (domains and exemplars). In addition, a discriminator is equipped during the training phase to guarantee the output satisfying the distribution of the target domain. Our EDIT can flexibly and effectively work on multiple domains and arbitrary exemplars in a unified neat model. We conduct experiments to show the efficacy of our design, and reveal its advances over other state-of-the-art methods both quantitatively and qualitatively.

## 1. Introduction

Visual scenes can be expressed in various manners using sketches, semantic maps, photographs, and painting artworks, to name just a few. Basically, the way that one portrays the scene and expresses his/her vision is the so-called style, which can reflect the characteristic of either a class/domain or a specific case. Image-to-image translation (I2IT) [1] refers to the process of converting an image *I* of a certain style to another target style $S_{t}$ with the content preserved. Formally, seeking a desired translator T can be written in the following form:

(1) $$\begin{array}{c} \min\;C\left(I_{t},I\right)\;+\;S\left(I_{t},S_{t}\right)\;\;\mathrm{with}\;\;I_{t}:=T\left(I,S_{t}\right), \end{array}$$

where $C(I_{t},I)$ is to measure the content difference between the translated $I_{t}$ and the original *I*, while $S(I_{t},S_{t})$ is to enforce the style of $I_{t}$ following that indicated by $S_{t}$.

### 1.1. Previous Work

With the emergence of deep techniques, a variety of I2IT strategies have been proposed with great progress made over the last decade. In what follows, we briefly review contemporary works along two main technical lines, i.e., one-to-one translation and many-to-many translation.

#### 1.1.1. One-to-One Translation

Methods in this category aim at mapping images from a source domain to a target domain. Benefiting from the generative adversarial networks (GANs) [2], the style of translated results satisfies the distribution of the target domain Y, achieved by $S\left(I_{t},S_{t}\right):=D\left(I_{t},Y\right)$, where $D(I_{t},Y)$ represents a discriminator to distinguish if $I_{t}$ is real with respect to Y. An early attempt by Isola et al. [1] uses conditional GANs to learn mappings between two domains. The content preservation is supervised by the paired data, i.e., $C\left(I_{t},I\right):=C\left(I_{t},I_{t}^{gt}\right)$ with $I_{t}^{gt}$ the ground-truth target. However, in real-world situations, acquiring such paired datasets, if not impossible, is impractical. To alleviate the pressure from data, inspired by the concept of cycle consistency, cycleGAN [3], DualGAN [4], DiscoGAN [5] in an unsupervised fashion were proposed, which adopt $C\left(I_{t},I\right):=C\left(F_{Y\to X}\left(F_{X\to Y}\left(I\right)\right),I\right)$ with $F_{X\to Y}$ the generator from domain X to Y and $F_{Y\to X}$ the reverse one. Afterwards, many works [6,7,8,9] further extend the translation between two domains to that cross multiple domains in a single model. Though the effectiveness of the mentioned methods has been witnessed by a wide spectrum of specific applications such as photo-caricature [10,11], make-up removal [12], and face manipulation [13], their main drawback comes from the nature of deterministic (uncontrollable) one-to-one mapping.

#### 1.1.2. Many-to-Many Translation

**Figure 1:**
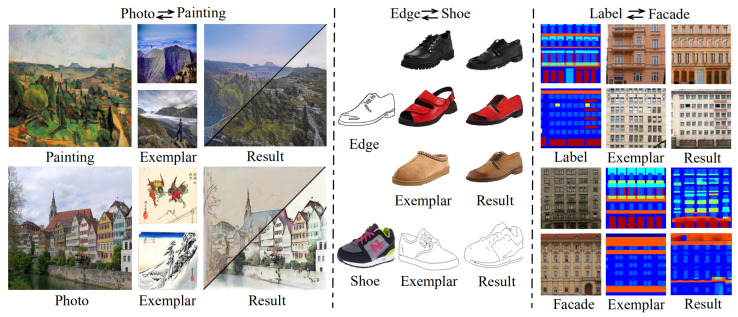
Several results by the proposed EDIT. Our EDIT is able to take arbitrary exemplars as reference for translating images across multiple domains including photo-painting, shoe-edge, and semantic map-facade in one model.

The goal of approaches in this class is to transfer the style controlled by an exemplar image to a source image with content maintained. Arguably, the most representative work goes to [14], which uses the pre-trained VGG16 network [15] to extract the content and style features, then transfer style information by minimizing the distance between Gram matrices constructed from the generated image and the exemplar *E*, say $S\left(I_{t},S_{t}\right):=S\left(\mathrm{Gram}\left(I_{t}\right),\mathrm{Gram}\left(E\right)\right)$. Since then, numerous applications on 3D scene [16], face swap [17], portrait stylization [18] and font design [19] have been done. Furthermore, a number of schemes have also been developed towards relieving the limitations of [14] in terms of speed and flexibility. For example, to tackle the requirement of training for every new exemplar (style), Shen et al. [20] built a meta-network, which takes in the style image and produces a corresponding image transformation network directly. Risser et al. [21] proposed the histogram loss to overcome the training instability. Huang and Belongie [22] designed a more suitable normalization manner, i.e., AdaIN, for style transfer. Li et al. [23] replaced the Gram matrices with an alternative distribution alignment manner from the perspective of domain adaption. Johnson et al. [24] trained the network with a specific style image and multiple content images while keeping the parameters at the inference stage. Chen et al. [25] introduced a style-bank layer containing several filter-banks, each of which represents a specific style. Gu et al. [26] proposed a style loss to make parameterized and non-parameterized methods complement each other. Huang et al. [27] designed a new temporal loss to ensure the style consistency between frames of a video. In addition, to consider the style of a domain/class, several works, for instance [28,29,30], advocated to separately take care of domain invariant content c(I) and domain-specific style s(I) subject to I≃c(I)∘s(I) with ∘ the combination operation. They manage to control the translated results by combining the content of an image with the style of the target domain, i.e., c(I)∘s(E). Besides their performance is inferior to our method in visual quality, diversity, and style preservation as observed from comparisons, they have two main weaknesses: one is that a domain pair usually requires an independent model; another is that their exemplars are constrained to be from the target domains. Please see Figure 1 for images generated by our method that handles multiple domains and arbitrary exemplars in a unified model.

### 1.2. Challenges and Motivations

Developing a practical I2I translator remains challenging because the capabilities of preserving content information after translation, and handling multiple domains as well as arbitrary exemplars should be considered jointly. We list the challenges as follows:

- How to rationally disentangle the content and style representations of images from different domains in a unified fashion (multi-domain in one model)?
- How to effectively ensure the content of the translated result being consistent with that of the original image in an unsupervised manner (content preservation)?
- How to flexibly manipulate an image by considering both the style of a target domain and that of a specific exemplar (exemplar-domain style awareness)?

Our principle is that, for images from different domains, the content features can be obtained by an explicit uniform extractor, while (re-)stylization is achieved by mapping the extracted features specifically to different purposes. We note that the network size can be significantly reduced through logical network partition, which will be verified in Section 3. This principle is rational: taking artwork composition for example, given a fixed scene, the physical content is the same, but the styles of presentation can be much diverse by different artists. For the style factor, one may generally like the paintings by Monet (domain), and among so many pieces of art, a particular one, e.g., “Water Lilies” (exemplar), is his/her favorite. In other words, the domain-level and exemplar-level should be simultaneously concerned during style transfer. Moreover, to maintain the content information after translation, the cycle consistency can be employed due to its effectiveness and simplicity. It is worth emphasizing that, a single generator instead of a pair, like cycleGAN [3], could be sufficient if the content and style are well-disentangled.

### 1.3. Contributions

Motivated by the above principle, we propose a novel network to overcome the mentioned challenges. Concretely, our primary contributions can be summarized as follows:

- We design a network, namely EDIT, to produce diverse results in an unsupervised controllable (many-to-many) fashion, which can flexibly and effectively work on multiple domains and arbitrary exemplars in a unified neat model.
- The generator of our EDIT comprises of a part of blocks configured by shared parameters to uniformly extract content features for images from multiple domains, and the rest by varied parameters exported by an exemplar-domain aware parameter network to catch specific style information.
- To preserve the content between input and generated result in an unsupervised manner, the cycle consistency is employed. Plus, a discriminator is equipped during the training phase to guarantee the output satisfying the distribution of the target domain.
- We conduct extensive experimental results to reveal the efficacy of our design, and demonstrate its advantages over other state-of-the-art methods both quantitatively and qualitatively.

Several previous works, with feature transform in [31], style decoration in [32], and feature normalization transfer in [33] as representatives, insert an extra step, say feature manipulation, between the trained encoder and decoder to achieve style transfer, the spirit of which is seemingly similar but much different to ours. We achieve the style transfer in the decoder dynamically generated. Even if there is no exemplar provided, the model still can produce results according to the target domain (by setting the exemplar to e.g., a black image or a noise image), which is more flexible than traditional style transfer methods like [31,32] having no domain information considered. Please notice that although DRIT [28], cd-GAN [29], MUNIT [30] and EGSC-IT [33] achieve exemplar guided many-to-many translation, they require to independently train different models for different domain pairs, and constrain the exemplars to be inside the target domains. Specifically, they introduce two encoders for each domain (four encoders for a pair of domains), to respectively extract the domain invariant content c(·) and domain specific style s(·) from an image, respectively. The combination of the content c(I) from an image *I*, and the style s(E) from an exemplar *E*, i.e., c(I)∘s(E), is the I2I translation result. The domain-specific style/information of these methods can only be extracted from the exemplar image, say the style of a domain and an exemplar are not well-decoupled, resulting in their exemplar images must be inside the target domains. In comparison, our method is able to embrace multiple domain pairs and arbitrary exemplars in one neat model.

## 2. Methodology

### 2.1. Problem Analysis

A desired translator should be capable to generate diverse results in a controllable (many-to-many) fashion. Again, we emphasize the core principle behind this work: for images from different domains, the content features can be obtained by an explicit uniform extractor, while (re-)stylization is achieved by mapping the extracted features specifically to different purposes. In other words, we assume that the content c(·) and the style s(·) of an image are independent, i.e., p(I)=p(c(I),s(I))=p(c(I))·p(s(I)). Suppose that the whole style space is ${⋃}_{i}S_{i}$, where $S_{i}$ is the style subspace corresponding to the domain *i*. Mathematically, the problem can be expressed and solved by maximizing the following probability:

(2) $$\begin{array}{cc} p(I_{x}^{y} & |I_{x},I_{y}):=p\left(c\left(I_{x}^{y}\right),s\left(I_{x}^{y}\right)|c\left(I_{x}\right),s\left(I_{x}\right),c\left(I_{y}\right),s\left(I_{y}\right)\right)\\ \; & \propto p\left(c\left(I_{x}^{y}\right)|c\left(I_{x}\right)\right)\cdot p\left(s\left(I_{x}^{y}\right)|s\left(I_{y}\right)\right)\\ \; & =p\left(c\left(I_{x}^{y}\right)|c\left(I_{x}\right)\right)\cdot \sum_{i}p\left(s\left(I_{x}^{y}\right)|s\left(I_{y}\right),S_{i}\right)\cdot p\left(S_{i}\right)\\ \; & =\sum_{i}p\left(c\left(I_{x}^{y}\right)|c\left(I_{x}\right)\right)\cdot p\left(s\left(I_{x}^{y}\right)|s\left(I_{y}\right),S_{i}\right)\cdot p\left(S_{i}\right). \end{array}$$

**Figure 2:**
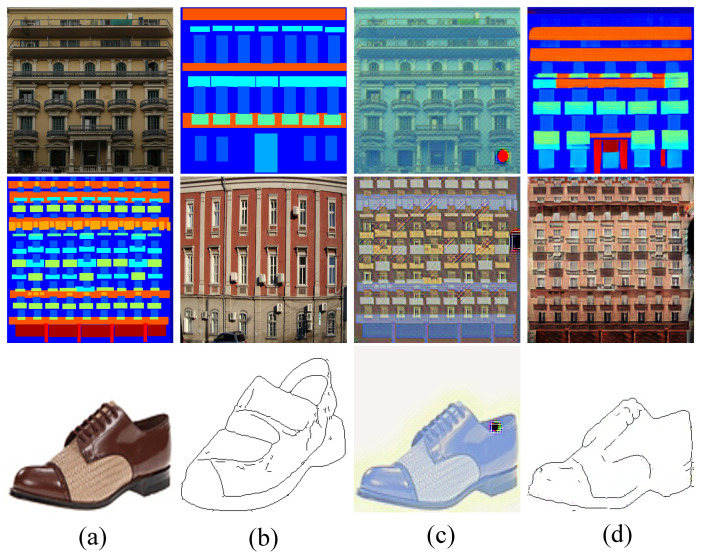
Visual results by EDIT with and without specifying the target domain. Panels (**a**,**b**) contain the inputs $I_{x}$ and exemplars $I_{y}$, respectively. Panels (**c**,**d**) give the translated results without and with domain specification, respectively.

The relationship of the second row holds by the problem definition in Equation (1) and the independence assumption (our core principle). Furthermore, the style of $I_{y}$ may appear in more than one domains, for instance, a semantic map can also be a painting. This situation makes $p(s\left(I_{x}^{y}\right)|s\left(I_{y}\right))$ a mixture of $\sum_{i}p\left(s\left(I_{x}^{y}\right)|s\left(I_{y}\right),S_{i}\right)\cdot p\left(S_{i}\right)$ (the equality of the third row). Please see Figure 2 for evidence. Therefore, we specify the domain label to clear the mix-up. By doing so, the problem turns to maximize the following:

(3) $$\begin{array}{cc} p(I_{x}^{y\in S_{i}}|I_{x},I_{y} & \in S_{i})\\ := & p\left(c\left(I_{x}^{y}\right)|c\left(I_{x}\right)\right)\cdot p\left(s\left(I_{x}^{y}\right)|s\left(I_{y}\right),S_{i}\right). \end{array}$$

As given in Equation (3), the entire problem can thus be divided into two subproblems. The first component $p(c\left(I_{x}^{y}\right)|c\left(I_{x}\right))$ corresponds to the uniform content extractor, while the second term $p(s\left(I_{x}^{y}\right)|s\left(I_{y}\right),S_{i})$ yields the exemplar-domain aware style mapping. The above logical partition strategy decomposes the parameter space, and thus expecting the reduction in storage and training costs.

### 2.2. Architecture Design

**Figure 3:**
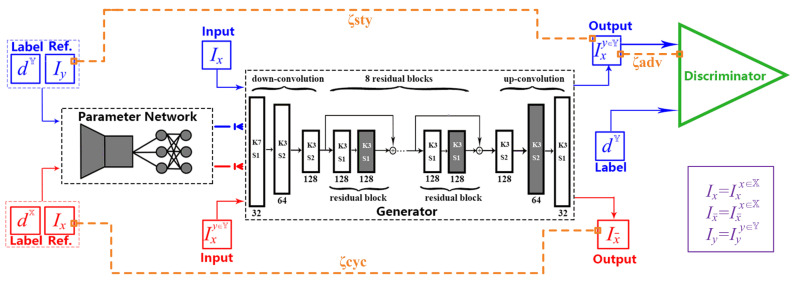
The model architecture of our EDIT. The procedure of mapping X→Y is in blue, while the reverse of mapping Y→X is in red. $I_{x}$ and $I_{y}$ are samples from domain X and Y, respectively. The whole network comprises a generator and a discriminator. The generator contains a part of blocks configured by shared parameters, and the rest by varied parameters exported by an exemplar-domain-aware parameter network. The parameter network generates the specific parameters based on an exemplar and its domain label. The content is preserved by adopting the cycle consistency. The discriminator takes a generated result and its domain label as input to judge if the result is distinguishable from the target domain. $K_{k}$ means that the kernel size is k×k, while $S_{s}$ represents that the stride is *s*. The number of channels is given below each block.

The blueprint of our EDIT is schematically illustrated in Figure 3, from which, we can see that the generator G of EDIT is composed by a part of blocks configured by shared parameters $\theta_{s}$, and the rest by varied parameters $\theta_{p}$ exported by an exemplar-domain aware parameter auxiliary network. In addition, a discriminator D is equipped during the training phase to guarantee the output satisfying the distribution of the target domain.

The generator is used to produce desired images through

(4) $$I_{x}^{y\in S_{i}}:=G\left(I_{x},I_{y}\in S_{i};\theta \right),$$

where θ is the trainable parameters for the whole generator. The generator consists of three gradually down-sampled encoding blocks, followed by 8 residual blocks. Then, the decoder processes the feature maps gradually up-sampled to the same size as input. Each block performs in the manner of Conv + InstanceNorm + ReLU. As stated, a part of the generator should respond to extract features uniformly for images no matter what styles they are in. In other words, a number of blocks (in white as shown in Figure 3, uniform content extractor) are shared across domains, the parameter set of which is denoted by $\theta_{s}$. As for the rest blocks (in black as shown in Figure 3) related to (re)-stylization (feature selection and reassembling). Inspired by [20,34], the corresponding parameters can be dynamically generated by a parameter network, that is:

(5) $$\theta_{p}:=G_{P}\left(I_{y}\in S_{i};\psi \right),$$

where ψ is its trainable parameters. Please notice that our parameter network only covers a part, instead of all of the blocks in the generator as [20,34], which significantly saves the storage space and increases the convergence speed. Specifically, the parameter network contains the VGG16 network pre-trained on the ImageNet and fixed, followed by one fully-connected layer and one group fully connected layer. Feeding an exemplar (style image) and its target domain label (a one-hot vector) into the parameter network gives the parameters required by the exemplar-domain aware style mapping. Now, we can express the generator in the following shape:

(6) $$I_{x}^{y\in S_{i}}:=G\left(I_{x};\theta_{p}:=G_{P}\left(I_{y}\in S_{i};\psi \right),\theta_{s}\right),$$

where both $\theta_{s}$ and $\theta_{p}$ form θ.

Based on the analysis on domain specification, it is important to clear the style mix-up issue as revealed in Figure 2. Merely providing the domain ID to the parameter network is insufficient to capture the domain characteristic, as it is blind to the distribution of the target domain. To guide the training process and produce high-quality images satisfying the distribution of the target domain, we further employ a discriminator built upon the 70×70 Patch-GAN architecture [35], which tries to determine whether each local image patch, rather than the whole image, is real or fake. More details about the discriminator can be found in the corresponding paper or in Appendix A. It is worth noting that the exemplar-domain aware style mapping is actually achieved by the dynamic part in the generator together with the discriminator.

One may wonder why inserting dynamic (black) blocks into fixed (white) blocks. First, considering the generation of dynamic parameters, the complexity of the fully connected layers will dramatically grow as the number of dynamic parameters (e.g., all the blocks in the decoder) required to generate increases. From another point of view, style mapping can be viewed as a procedure of feature selection and reassembling. Some operations should be in common for features from different domains. Taking the above concerns, we adopt the organization fashion as shown in Figure 3, which performs sufficiently well in practice and makes the volume of the parameter network compact. The primary merit of our EDIT is that it can handle arbitrary exemplars and be trained for multiple domains at the same time in one neat model. More details of EDIT are given in Appendix A.

### 2.3. Loss Design

We adopt a combination of a cycle consistency loss, a style loss and an adversarial loss for training the network.

**Cycle consistency loss.** Taking a sample pair $I_{x}\in X$ and $I_{y}\in Y$ for an example, let $I_{\bar{x}}$ and $I_{\bar{y}}$ be $G(G\left(I_{x},I_{y}\in Y\right),I_{x}\in X)$ and $G(G\left(I_{y},I_{x}\in X\right),I_{y}\in Y)$, respectively. To preserve content between generated and original images, the cycle consistency loss is employed, which is written as:
  (7) $$\begin{array}{c} \zeta_{cyc}:=∥I_{\bar{x}}-I_{x}{∥}_{1}\;+\;{∥I_{\bar{y}}-I_{y}∥}_{1}, \end{array}$$
  where ${∥\cdot ∥}_{1}$ is the $\ell_{1}$ norm.
**Style loss.** For allowing users to control the style by giving an exemplar, a measurement for style difference is required. As advocated in [23], the batch normalization statistics based loss is adopted instead of the Gram matrix based one, for ease of computation. By denoting $I_{\hat{x}}:=G\left(I_{x},I_{y}\in Y\right)$ and $I_{\hat{y}}:=G\left(I_{y},I_{x}\in X\right)$, we have:
  (8) $$\begin{array}{cc} \zeta_{sty}:= & \sum_{l=1}^{N_{L}}\sum_{m=1}^{M_{l}}\frac{\left({\left(\mu_{\hat{y}}^{l,m}-\mu_{x}^{l,m}\right)}^{2}\;+\;{\left(\sigma_{\hat{y}}^{l,m}-\sigma_{x}^{l,m}\right)}^{2}\right)}{N_{L}\times M_{l}}\\ \;+\; & \sum_{l=1}^{N_{L}}\sum_{m=1}^{M_{l}}\frac{\left({\left(\mu_{\hat{x}}^{l,m}-\mu_{y}^{l,m}\right)}^{2}\;+\;{\left(\sigma_{\hat{x}}^{l,m}-\sigma_{y}^{l,m}\right)}^{2}\right)}{N_{L}\times M_{l}}, \end{array}$$
  where $N_{L}$ and $M_{l}$ are the number of involved layers (in this work, we use the relu1_2, relu2_2, relu3_3, relu4_3 and relu5_1 layers in the VGG16) and that of feature maps in the *l*-th layer. In addition, μ and σ are the mean and the standard deviation of the corresponding feature map.
**Adversarial loss.** The adversarial loss is standard [2] as:
  (9) $$\begin{array}{cc} \zeta_{adv}:= & \log D\left(I_{x},X\right)\;+\;\log\left(1-D\left(G\left(I_{x},I_{y}\in Y\right),Y\right)\right)\\ \;+\; & \log D\left(I_{y},Y\right)\;+\;\log\left(1-D\left(G\left(I_{y},I_{x}\in X\right),X\right)\right). \end{array}$$
**Final objective.** Our optimization is carried out on the total loss, i.e., the sum of the above losses, as follows:
  (10) $$\begin{array}{cc} \; & \min_{G}\max_{D}\;\;E_{I_{x}∼P_{data}\left(X\right)}E_{I_{y}∼P_{data}\left(Y\right)}\;\;\zeta_{total},\\ \; & \mathrm{where}\;\;\zeta_{total}:=\zeta_{adv}\;+\;\lambda \zeta_{cyc}\;+\;\eta \zeta_{sty}, \end{array}$$
  where η and λ are coefficients to balance the loss terms. In order to keep the common features effectively, we set λ to a relatively large value 10. As for η, we observe that setting it in the range from 0.01 to 0.1 works well.

## 3. Experimental Validation

### 3.1. Implementation Details

Our EDIT is implemented in PyTorch and performed on a GeForce RTX 2080Ti GPU. We exert the strategy proposed in [36] to improve the training stability, which uses historic generated images to update the parameters of the discriminator. Our optimization adopts an Adam solver. The decays of the first and second-order momentums are as default. The learning rate is set to 0.001 at the beginning and linearly decreases as the number of epochs grows. During the training phase, the input images are resized to 256×256 and augmented by random horizontal flip.

### 3.2. Competitors and Evaluation Metrics

The competitors involved in comparisons contain neural style transfer (NST) [14], cycleGAN [3], metaNST [20], DRIT [28], MUNIT [30], WCT [31], EGSC-IT [33], and art2real [37]. The codes of the compared methods are all downloaded from the authors’ websites. The elapsed time of testing, model size, inception score (IS) [38] and Fréchet inception distance (FID) [39] are employed as our metrics to quantitatively reveal the performance difference between our EDIT with the other competitors. In addition, to measure how well the content and style are preserved, by following [14], the content error and style error are also adopted. Take the mapping: X→Y as an example, the content error $E_{cont}$ is defined as the L2 distance between feature maps of the input image $I_{x}$ and the generated one $I_{x}^{y\in Y}$, i.e., $\left|\right|\phi_{l}\left(I_{x}\right)-\phi_{l}\left(I_{x}^{y\in Y}\right){\left|\right|}_{2}$, where $\phi_{l}\left(\cdot \right)$ means the feature maps of the *l*-th layer in the VGG-16 model. The style error is the average L2 distance between the Gram matrices of the generated image $I_{x}^{y\in Y}$ and the exemplar $I_{y}$. Assume ${\mathrm{Gram}}_{l}\left(\cdot \right)$, $H_{l}$, and $W_{l}$ are the Gram matrix, height and width of the each feature map in the *l*-th layer, the style error can be expressed as $\frac{1}{N_{L}}\sum_{l=1}^{N_{L}}\frac{1}{4M_{l}^{2}H_{l}^{2}W_{l}^{2}}\left|\right|{\mathrm{Gram}}_{l}\left(I_{y}\right)-{\mathrm{Gram}}_{l}\left(I_{x}^{y\in Y}\right){\left|\right|}_{2}^{2}$. It is worth noticing that the content error and the style error are complement and expected to be a trade-off. Solely focusing on one of the two metrics cannot practically reflect the performance of I2I translation. For instance, a case without any change (style transfer) obtains 0 content error. Analogous problem happens to only staring at the style loss without consideration of content preservation.

### 3.3. Comparisons

To quantitatively measure the performance of different competitors, we conduct the experiments on the translation from photo to painting (Monet). The training and testing data are from [3]. The competitors are well-trained on the training data, and tested on the 750 testing data of the photoset with 10 exemplars from the Monet set. This is to say, each compared model generates 7500 images, on which the content error, the style error, the inception score and the Fréchet inception distance are computed.

From Table 1, we can observe that in terms of the content error, our EDIT slightly falls behind cycleGAN, while outperforms the others. The analogous analysis serves the inception score and Fréchet inception distance terms. We again emphasize that there exists a trade-off between the content and the style consistency in I2I translation. Notice that cycleGAN pays more attention to the content loss while only guaranteeing the domain style without the consideration of exemplars. As for the style loss, EDIT takes first place among all the compared methods with a large margin. The cycleGAN is unable to take exemplars as references. Thus, we do not provide its style error. In terms of model size, we provide two sets of comparisons: one for one pair domain translation and the other for n=4 pairs. Most of the methods including MUNIT, cycleGAN, DRIT, and EGSC-IT require training multiple independent models to handle multiple pairs of the domain. While EDIT can deal with multiple domain pairs in one model, thanks to either the dynamic parameter generators according to exemplars. The dynamic parameter generators for both EDIT and metaNST have several fully connected layers, making their models relatively large. A large part of the parameters in metaNST (10 Mb vs. 64 K for shared part) is from the parameter generator, leading to an 870 Mb storage. Ours demands the parameter network to produce about 2.9 Mb (vs. 3.3 Mb for the shared part) parameters dynamically, significantly decreasing the storage (409 Mb) compared with metaNST. In addition, different from NST, metaNST and WCT, EDIT further takes into account the domain knowledge/style and the cycle consistency. The above verifies, as previously stated in our principle, that the feature extraction can be done using a uniform extractor and part of feature reassembling can also be in common for different images from different domains. In this work, we consider four domain pairs, but it is possible to embrace more pairs in our EDIT.

**Figure 4:**
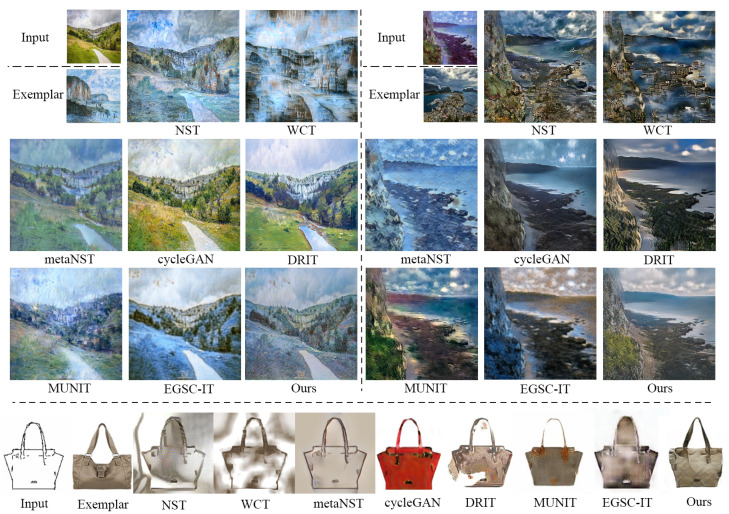
Visual comparison among the competitors on photo to painting, painting to photo, and edge to handbag.

**Figure 5:**
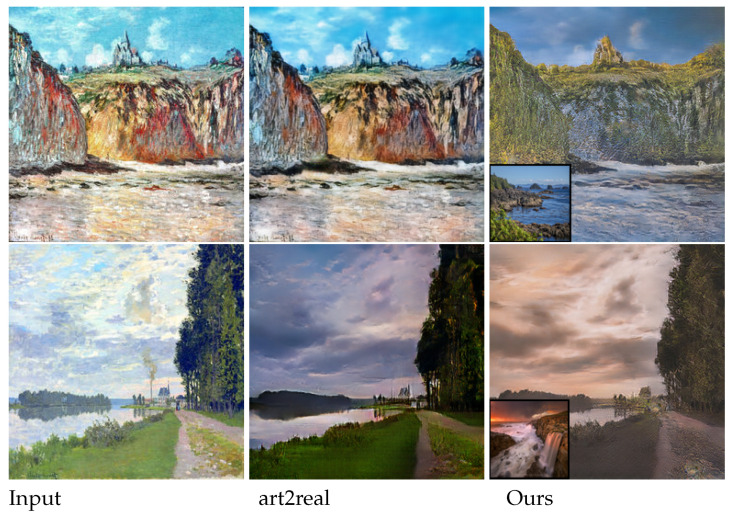
Visual comparison between art2real and EDIT.

**Figure 6:**
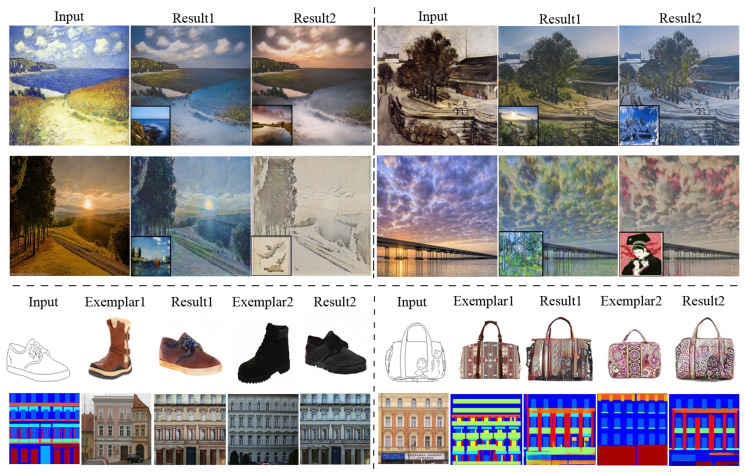
More visual results by our proposed EDIT.

In terms of speed, NST takes a much longer time, i.e., about 420 s to process a case with a size of 256×256, than the others, due to its processing way. The fastest method goes to cycleGAN (3.5 ms), as it does not need to consider exemplars. Among the methods that consider exemplars, our EDIT is the most efficient one (4.3 ms), slightly slower than cycleGAN. In addition, WCT is relatively slower (1.7 s) because of the requirement of SVD operation that has to be executed in CPU. In Table 1, we also report the numbers corresponding to EDIT with the discriminator disabled, which reveals the importance of the adversarial mechanism for the target task. Figure 4 depicts three visual comparisons to qualitatively show the difference among the competitors. From the pictures, we can see that our EDIT can very well preserve the content of input and transfer the style of exemplar, making the final results visually striking. It is worth noting that art2real is specifically designed for translation from arts/paintings to realistic photos without using any exemplar, which if reasonably modified, needs multiple models for different domain pairs in nature. Figure 5 additionally gives a comparison between art2real and EDIT. The result by art2real indeed has some features of paint removed, however, the unnatural-looking of which is still obvious. While, by taking an exemplar into consideration, EDIT produces a more realistic result. We provide other visual results by EDIT on painting ↔ painting, edge → shoe, edge → handbag, and semantic map ↔ facade in Figure 6. More results can be found at https://forawardstar.github.io/EDIT-Project-Page/ accessed on 30 April 2021. Our code is made publicly available at https://github.com/ForawardStar/EDIT accessed on 30 April 2021. Comprehensively, the proposed EDIT is arguably the best candidate.

### 3.4. Ablation Study

#### 3.4.1. Alternatives of the Dynamic Parameters

**Figure 7:**
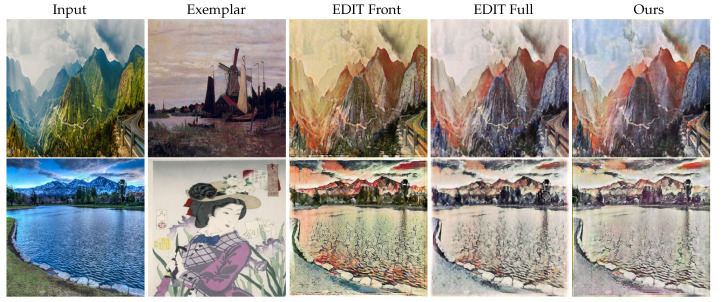
The comparison between various alternatives of the generated blocks. The 1st and 2rd rows are photos → Monet and photo → Ukiyoe, respectively.

There are various alternatives with respect to which part of parameters in the generator are dynamically generated by the parameter network or uniformly trained. We further conduct several experiments on different domain pairs to study the effects of two additional new settings of the generated blocks, including (1) the parameters of first-half blocks are generated by the parameter network while the rest parameters are uniformly trained (denoted as EDIT Front), and (2) the whole set of parameters are generated (EDIT Full). The visual results are shown in Figure 7, while the quantitative results are shown in Table 2.

**Figure 8:**
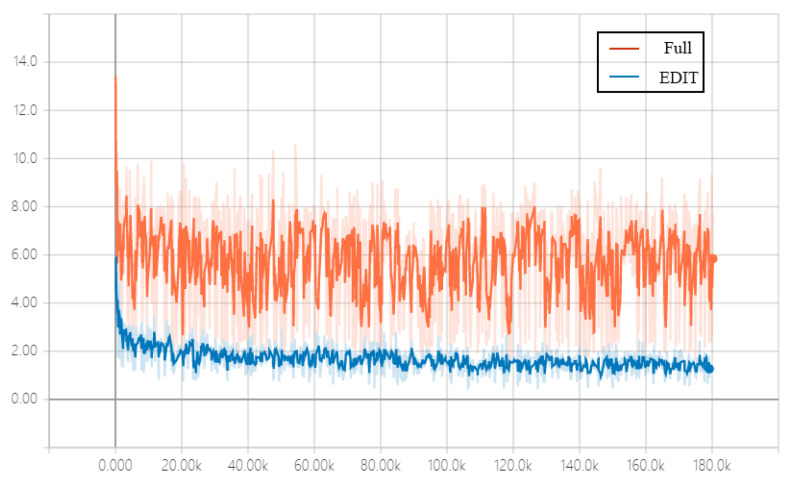
The comparison in convergence behavior of EDIT Full and EDIT.

It is obvious that in terms of the style error, EDIT Front performs worse than EDIT and EDIT Full, indicating that the parameters in shallow layers of the generator are more about extracting the content features of input images, which can be shared across domains, while the deeper layers are used to map the style features, which need to be dynamic with respect to domains and exemplar images. The performance of EDIT is competitive to that of EDIT Full proves that generating a part of, rather than all of, parameters of the generator is sufficient, as numerically and visually reported in Table 2 and Figure 7, respectively. In addition, EDIT Full is of much higher complexity than EDIT (1143 Mb vs. 409 Mb in size of the parameter network), and is unstable at the training stage, leading to slow convergence. The convergence behavior of EDIT Full and EDIT is shown in Figure 8.

#### 3.4.2. Alternatives of the Network Architecture

**Figure 9:**
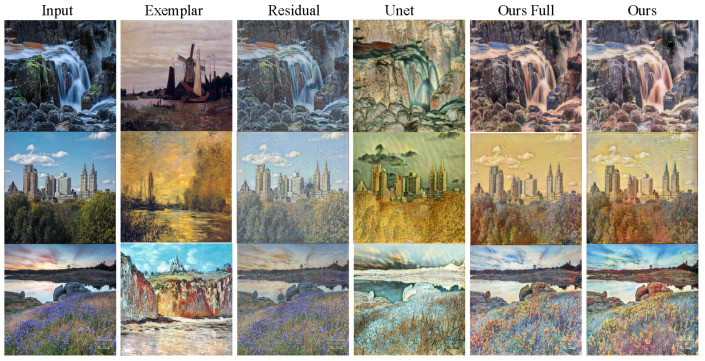
The comparison between various alternatives of the network architectures. All the rows are photo → Monet.

We compare our original architecture with two other alternatives. One is an Unet architecture adding skip connections to the original encoder–decode architecture, the other is using original architecture for residual learning whose output is added to the input image to get translated results. The results are shown in Table 1 and Figure 9. It can be seen that adding skip connections to an encoder–decoder leads to worse results. The reason is, directly feeding the low-level features extracted by the shallower layers into the deeper layers may disturb (high-level) style mapping operation. While the residual learning strategy tends to maintain the original input, since the generator merely learns the residual between input and translated results. Therefore, the residual learning strategy may be suitable for low-level tasks like denoising, dehazing and image smoothing, but not for the task of image-to-image translation/style transfer.

### 3.5. Extensions

In this section, we demonstrate that there are several extensions including style interpolation, and using exemplars outside the domains. The network architectures for these extensions are the same as the original EDIT, but the training and testing strategies are changed accordingly.

#### 3.5.1. Style Interpolation

**Figure 10:**
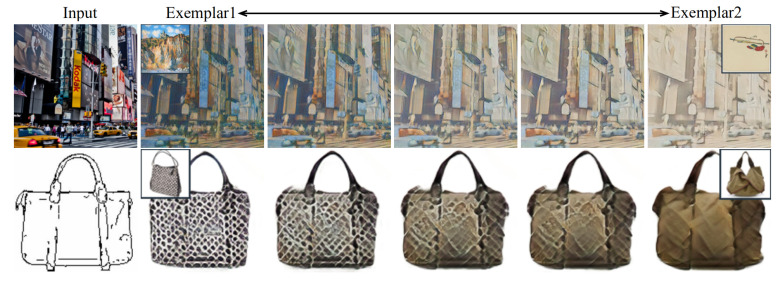
Interpolation results. The left-most column contains two inputs. The second and right-most columns are the results with respect to two different exemplars. The three columns in the middle are the interpolated results by EDIT.

One may want to take two or more exemplars/domains as a style reference, and produce results simultaneously containing those styles in a controllable fashion. Considering that the dynamic parameters correspond to the exemplars, they can be viewed as their representations in the implicit manifold. Suppose the manifold is continuous and smooth, we can linearly combine the generated parameters to achieve the style interpolation. Figure 10 displays two cases of style interpolation. The second and the sixth columns offer the translated results by fully using different exemplars. The pictures shown in the middle columns are results by linearly interpolating the parameters of the second and the last columns. As can be seen, via controlling the parameter combination, the visual results vary smoothly between two styles with the content well-preserved.

#### 3.5.2. Exemplars Outside the Domains

**Figure 11:**
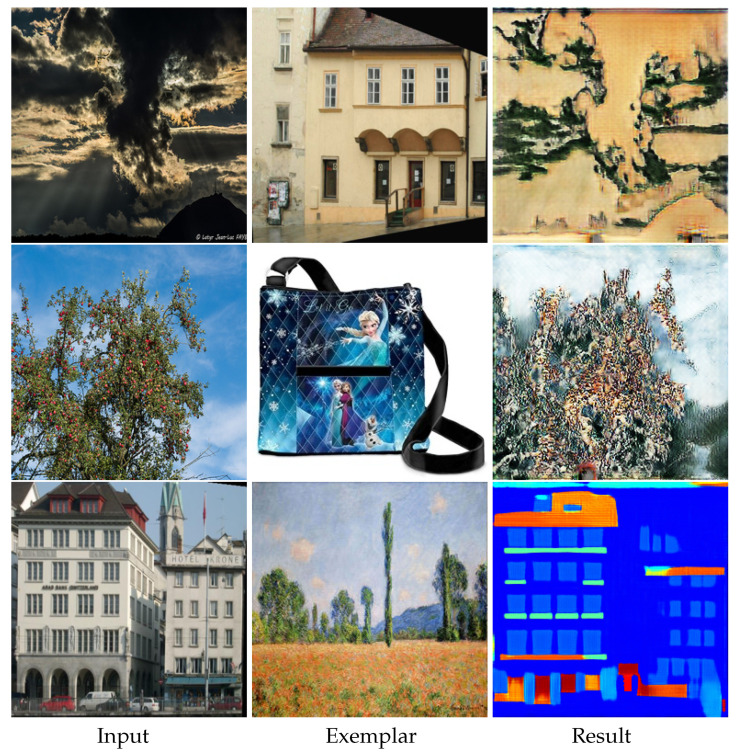
The results of using the exemplar images whose domains are different from the target domain. The 1st, 2nd, 3rd and 4th rows are photo → Ukiyoe with the facade as an exemplar, photo → Monet with the handbag as an exemplar, and facade → semantic map with Monet’s painting as an exemplar, respectively.

**Figure 12:**
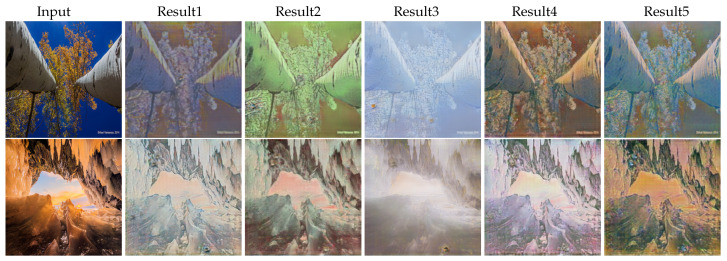
The results of replacing exemplar images with noises. The 1st and 2rd rows are both photo → Monet.

As an image may belong to more than one domain, we design our EDIT to use arbitrary exemplar images for multiple target domains, even the label/ID of the exemplar image and the target domain mismatch and the domains of the exemplar images are unknown or unseen during training. The results are shown in Figure 11, from the first two cases, we can see that if the exemplar falls in the domain distribution’s tail, EDIT still tries to produce results taking into account both domain and exemplar information. However, when the exemplars are totally outside of the target domain, EDIT will ignore the exemplars and rely on the domain only, as shown in the last case of Figure 11. Note that [28,29,30,33] cannot handle such cases, since they do not decouple the style of exemplars and domains, and must extract the domain-specific information from the exemplars. Furthermore, one may simply want to gain diverse translated results from one input image without any exemplar. Since we use a one-shot vector as domain label/ID instead of exemplar images to control the target domain, we can replace the exemplar images with random Gaussian noises to generate diverse results as shown in Figure 12.

## 4. Conclusions

In this paper, we have proposed a network, called EDIT, to translate images from different domains with consideration of specific exemplars in a unified model. The generator of EDIT is built upon a part of blocks configured by shared parameters to uniformly extract features for images from multiple domains, and another part by dynamic parameters exported by an exemplar-domain aware parameter network to catch specific style information. The concepts of cycle consistency and adversarial mechanism make the translation preserve the content and satisfy the distribution of the target domain. Both theoretical findings and experimental results are provided to demonstrate the efficacy of the proposed framework. The quantitative experimental results demonstrate that our EDIT only takes less than five milliseconds to process a 256×256 image on a GTX 2080Ti GPU, and improve the image-to-image translation performance by around 5% in terms of inception score and Fréchet inception distance. We have also conducted extensive experiments to reveal the superiority of our method over other state-of-the-art alternatives. 

## Figures and Tables

**Table 1:** Quantitative comparison with the state-of-the-art methods. The two columns of model size (parameters) are for one domain pair and *n* domain pairs, respectively. For the Fréchet inception distance, content and style error, lower value indicates better performance. While for the inception score, the higher the better.

Methods	Time (sec.)	Paras. (Mb|1 pair)	(*n* = 4 pairs)	Content Error ↓	Style Error ↓	IS ↑	FID ↓
cycleGAN [3]	$3.5\times 10^{-3}$	45 + 45	(45 + 45)×n	1.70 ± 0.60	−	6.02	70.37
MUNIT [30]	$3.9\times 10^{-2}$	114.7	114.7×n	2.43 ± 1.28	0.19 ± 0.15	4.58	114.18
DRIT [28]	$1.2\times 10^{-2}$	780	780×n	2.83 ± 1.17	0.14 ± 0.09	5.06	80.07
NST [14]	$4.3\times 10^{2}$	576	576×n	3.43 ± 1.04	1.28 ± 0.56	5.85	83.63
metaNST [20]	$5.3\times 10^{-3}$	64K + 10 [+870]	64K + 10 [+870]	2.97 ± 0.93	0.13 ± 0.09	4.74	91.24
WCT [31]	$1.7\times 10^{0}$	283.6	283.6	4.92 ± 0.15	0.13 ± 0.07	3.09	89.73
EGSC-IT [33]	$8.2\times 10^{-1}$	135	135×n	2.71 ± 1.29	0.26 ± 0.19	5.50	75.65
EDIT w/o Adv	$4.3\times 10^{-3}$	3.3 + 2.9 [+409]	3.3 + 2.9 [+409]	3.39 ± 0.98	0.26 ± 0.10	4.59	107.98
EDIT Residual	$4.5\times 10^{-3}$	3.3 + 2.9 [+409]	3.3 + 2.9 [+409]	0.80 ± 0.20	0.33 ± 0.21	5.96	121.23
EDIT Unet	$4.7\times 10^{-2}$	3.2 + 4.1 [+612]	3.2 + 4.1 [+612]	5.47 ± 1.53	0.24 ± 0.14	5.84	95.24
**EDIT**	$4.3\times 10^{-3}$	3.3 + 2.9 [+409]	3.3 + 2.9 [+409]	2.60 ± 0.59	0.06 ± 0.04	5.92	72.48

**Table 2:** Quantitative comparison with various alternatives of the generated blocks. The first column is the corresponding dataset.

Datasets	Methods	Content Error ↓	Style Error ↓	IS ↑	FID ↓
photo	EDIT Front	2.48 ± 0.67	0.11 ± 0.10	5.94	74.29
↓	EDIT Full	2.44 ± 0.63	0.07 ± 0.05	5.97	69.37
Monet	EDIT	2.60 ± 0.59	0.06 ± 0.04	5.92	72.48
Monet	EDIT Front	3.34 ± 1.29	0.33 ± 0.35	3.48	99.57
↓	EDIT Full	3.39 ± 1.33	0.27 ± 0.31	3.55	89.67
photo	EDIT	3.55 ± 1.30	0.22 ± 0.27	3.49	78.39
photo	EDIT Front	2.58 ± 0.66	0.13 ± 0.08	5.02	84.11
↓	EDIT Full	2.48 ± 0.67	0.12 ± 0.07	4.87	80.55
Ukiyoe	EDIT	2.56 ± 0.68	0.09 ± 0.06	4.80	81.88
Ukiyoe	EDIT Front	3.21 ± 1.20	0.33 ± 0.42	3.51	127.89
↓	EDIT Full	3.27 ± 1.25	0.28 ± 0.37	4.00	124.98
photo	EDIT	3.52 ± 1.28	0.23 ± 0.31	3.56	106.91
label	EDIT Front	6.53 ± 1.42	0.85 ± 0.93	1.91	104.00
↓	EDIT Full	6.56 ± 1.33	0.83 ± 1.00	2.10	114.60
facade	EDIT	6.33 ± 1.52	0.78 ± 0.86	2.25	105.61
facade	EDIT Front	10.08 ± 1.89	3.72 ± 2.73	2.50	127.46
↓	EDIT Full	9.96 ± 2.05	4.01 ± 2.77	2.86	154.40
label	EDIT	12.30 ± 2.18	3.68 ± 2.55	2.91	115.57

## Data Availability

MDPI Research Data Policies at https://people.eecs.berkeley.edu/~taesung_park/CycleGAN/datasets/ accessed on 30 April 2021 and https://forawardstar.github.io/EDIT-Project-Page/ accessed on 30 April 2021.

## Appendix A. Network Details

### Appendix A.1. Parameter Network

Our parameter network includes the convolution layers of VGG16 followed by one fully connected layer and one group fully connected layer. The detailed architecture is shown in Table A1. The *c*, *k*, *s* and *p* stand for the channel number, the kernel size, the stride step and the padding size, respectively. The $N_{neuron}$, $N_{group}$, $N_{para}$ and $M_{para}^{l}$ in the bottom part are the number of neurons in a layer of each group, the number of group of a group fully-connected layer ($N_{group}$ = 1 if is not a group fully-connected layer), the number of layers in the generator whose parameters need to be generated and the number of parameters in the *l*-th layer where 1 ≤ *l*$\leq N_{para}$, respectively.

**Table A1:** The parameter network architecture.

Convolution Layers					
**Output Size**	**Operator**	**c**	***k***	***s***	***p***
256×256	Conv2d-ReLU	64	3	1	1
256×256	Conv2d-ReLU (relu1_2)	64	3	1	1
128×128	MaxPool2d	-	-	-	-
128×128	Conv2d-ReLU	128	3	1	1
128×128	Conv2d-ReLU (relu2_2)	128	3	1	1
64×64	MaxPool2d	-	-	-	-
64×64	Conv2d-ReLU	256	3	1	1
64×64	Conv2d-ReLU	256	3	1	1
64×64	Conv2d-ReLU (relu3_3)	256	3	1	1
32×32	MaxPool2d	-	-	-	-
32×32	Conv2d-ReLU	512	3	1	1
32×32	Conv2d-ReLU	512	3	1	1
32×32	Conv2d-ReLU (relu4_3)	512	3	1	1
16×16	MaxPool2d	-	-	-	-
16×16	Conv2d-ReLU (relu5_1)	512	3	1	1
**Intermediate Processing**					
**Input**		**Operation**		**Output Size**	
relu1_2,relu2_2,		calculate mean			
relu3_3,relu4_3		and standard		1 × 2944	
relu5_3		deviation			
**Fully-Connected Layers**					
**layer**		$N_{\mathrm{neuron}}$		$N_{\mathrm{group}}$	
1		128 $\times \;N_{para}$		1	
2		$M_{para}^{l}$		$N_{para}$	

### Appendix A.2. Generator

The generator has an encoder-decoder architecture. We notice that the more parameters needed to be generated using the parameter network, the larger the model size is required for the parameter network. As shown in Table A2, our generator begins with three down-convolution layers to extract and encode the features of input images. Afterwards, nine residual blocks are used to process the style-related features according to exemplars. Trainable indicates whether the layer is jointly trained with the parameter network (Trainable = True) or generated by the parameter network (Trainable = False).

**Table A2:** The generator architecture.

Name	Operator	Trainable	Repeat	c	*k*	*s*
down-conv	Conv2d-IN-ReLU	True	1	32	9	2
	Conv2d-IN-ReLU	True		64	3	2
	Conv2d-IN-ReLU	True		128	3	2
residual block	Conv2d-IN-ReLU	True	8	128	3	1
	Conv2d-IN-ReLU	False		128	3	1
up-conv	Upsample-Conv2d-IN-ReLU	True	1	128	3	1
	Upsample-Conv2d-IN-ReLU	False		64	3	1
	Conv2d-Tanh	True		32	9	1

### Appendix A.3. Discriminator

The discriminator adopts the architecture of 70 × 70 patchGAN, which owns the ability to generate high-quality and high-resolution images. The discriminator consists of four Conv2d-IN-LeakyReLU blocks followed by one zero pad layer and one Conv2d layer. The detailed architecture is given in Table A3.

**Table A3:** The discriminator architecture.

Output Size	Operator	c	*k*	*s*	*p*
128 × 128	Conv2d-LeakyReLU	64	9	2	1
64 × 64	Conv2d-IN-LeakyReLUReLU	128	3	2	1
32 × 32	Conv2d-IN-LeakyReLU	256	3	2	1
16 × 16	Conv2d-IN-LeakyReLU	512	3	2	1
17 × 17	ZeroPad2d	-	-	-	
16 × 16	Conv2d	1	4	1	1
